# Review of automatic continuous distraction osteogenesis devices for mandibular reconstruction applications

**DOI:** 10.1186/s12938-020-00761-8

**Published:** 2020-04-01

**Authors:** Shahrokh Hatefi, Katayoun Hatefi, Francis Le Roux, Javad Alizargar, Zeinolabedin Behdadipour, Yimesker Yihun, Khaled Abou-El-Hossein

**Affiliations:** 1 Precision Engineering Laboratory, Nelson Mandela University, Port Elizabeth, South Africa; 2 Department of Electrical and Computer Engineering, Isfahan University of Technology, Esfahan, Iran; 3 Department of Mechatronics Engineering, Nelson Mandela University, Port Elizabeth, South Africa; 4 Research Center for Healthcare Industry Innovation, National Taipei University of Nursing and Health Sciences, Taipei, 112 Taiwan; 5 School of Medicine, Isfahan University of Medical Sciences, Esfahan, Iran; 6 Robotics Laboratory, Department of Mechanical Engineering, Wichita State University, Wichita, USA

**Keywords:** Automatic continuous distractor, Oral and maxillofacial reconstruction, Distraction osteogenesis, Medical devices

## Abstract

Distraction osteogenesis (DO) is an emerging method for bone tissue reconstruction. In oral and maxillofacial reconstruction applications, DO is playing an important role as a technique without the need of bone graft. In addition, in a DO treatment procedure, a superior outcome could be achieved compared to conventional reconstruction techniques. Recently, a few automatic continuous distraction osteogenesis (ACDO) devices have been designed and developed to be used in human reconstruction applications. Experiments and animal studies have validated the functionality of the developed ACDO devices. It has shown that by using such ACDO devices in a DO procedure, compared to conventional manual DO methods, superior outcomes could be obtained. However, the application of such ACDO devices is still limited. More research and investigation need to be undertaken to study all requirements of ACDO devices to be used in successful human mandibular DO treatment. It is important to determine all requirements and standards that need to be considered and applied in the design and development of ACDO devices. The purpose of this review paper is to highlight the designed and developed ACDO procedures thus far in terms of their working principles, working parameters, and technical aspects for providing a better perspective of the development progress of ACDO devices for oral and maxillofacial reconstruction applications. In this paper, design principles, device specifications, and working parameters of ACDO devices are compared and discussed. Subsequently, current limitations and gaps have been addressed, and future works for enabling an ultimate automatic DO procedure have been suggested.

## Background

The method of distraction osteogenesis (DO) is a recently emerging reconstruction solution for bone tissue lengthening and reconstructing. By using DO, different bone defects, congenital growth retardation of the bone tissue, and skeletal deformities can be reconstructed [1–3]. In maxillofacial reconstruction applications (MRA), DO is known as a new solution for bone tissue reconstruction without the need of bone graft. Therefore, the DO method has received more attention among all solutions and it is the first choice in MRA. By using the DO bone lengthening technique, the bone generation happens along with the adoption of the surrounding soft tissue and more predictable results could be obtained [4–7]. Using DO could reduce complications and limitations of other reconstruction methods, including osteoinduction, allograft implantations, autologous bone graft, and osteoprogenitor [8–10].

**Fig. 1:**
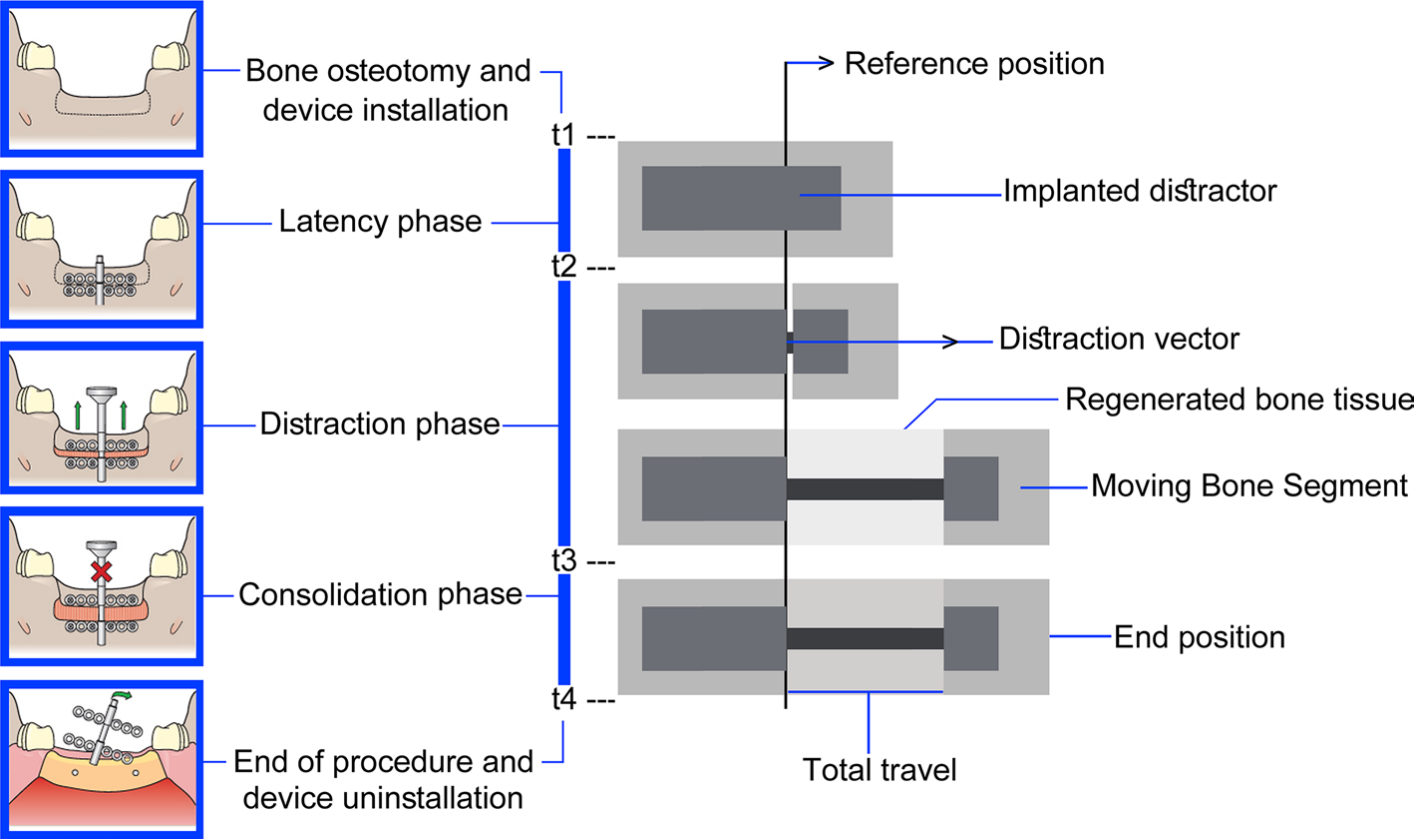
The standard DO protocol in MRA

In 1987 Ilizarov developed the DO technique and introduced this reconstruction method for MRA to scientific community. Consequently, in 1992 MacCarthy reported the first successful application of DO treatment on mandible [11–13]. In a typical DO procedure, a manual distractor is used. Figure 1 illustrates a standard DO protocol for MRA. A standard DO protocol consists of four phases: bone osteotomy and device installation, latency, activation, and consolidation [14–16]. As illustrated in Fig. 1, the DO procedure starts with the bone osteotomy and installation of the distractor (t1). In the next phase, called latency, the distractor is installed on the defected location without any activation (t1 to t2), while the osteogenic cells in the osteotomized location have already started regenerating and consolidating. After the latency, the activation phase begins and the moving bone segment (BS) moves through a predetermined linear path (distraction vector) towards the desired position to fulfill the defect (t2 to t3). After activation phase, there is a consolidation phase without activating the distractor (t3 to t4), and then by performing a second surgical procedure the device is removed (t4).

In the manual distractors, the activation and the movement of BS is upon manual activations by an operator. Two types of manual distractors have been developed: intra- and extra-oral. The extra-oral distractor was developed in 1987 by Ilizarov [11, 17]. There are major concerns and complications while using an extra-oral distractor; nerve injuries, scar formation, infection, and patient discomfort are from those serious complications that significantly influence the outcome of treatment and limit the application of such extra-oral devices, while, intra-oral distractors have shown better results during and after DO treatment [18, 19]. In both intra- and extra-oral distractors, the distractor is activated and the BS is moved once or twice a day towards the destination position, with a distraction accuracy of 0.5 to 1 mm. Published articles, reports, and clinical experiments have revealed the success of this novel technique. Manual distractors have been widely used in MRA. However, in manual distractors the activation of the device relies upon manual adjustment of length. There is a protentional error in manual length adjustment while applying an uncertain amount of distraction force (DF) for executing the movement. The major limitations of manual distractors are unstable movement, large step accuracy, low distraction rate (DR) from 0.25 to 1 mm/day, and low distraction rhythm (once or twice daily). Long treatment period, scar formation, painful distraction phase, and patient compliance are other issues in manual solutions [16, 20–24].

Further studies have proved that during a DO treatment, during the activation phase, by increasing the activation sequences for distracting and moving the BS (called distraction rhythm), superior results in bone regeneration and consolidation phases, as well as, a faster treatment period could be obtained [14, 25, 26]. The advantages of using quasi-continuous methods with higher rhythms of distraction have lead researchers and scholars to focus on design and development of automatic continuous distraction osteogenesis (ACDO) devices. In an ACDO treatment, an automatic system is implemented for producing a continuous DF and moving the BS towards the destination position in a predetermined linear/nonlinear path. ACDO devices have a very high distraction accuracy compared to conventional manual distractors; therefore, in an ACDO treatment the DR and rhythm could significantly be increased. Using higher DR with reduced distraction steps in the activation phase would result in a shorter treatment period and reduce patient discomfort and pain while improving the outcome of the treatment. Recent animal studies by using automatic continuous distractors have verified the viability of using an ACDO device in a DO treatment; histologic, radiographic, and samples of bone tissue have proved that using an ACDO solution can lead to a more successful treatment compared to manual solutions. Using continuous DF in a DO protocol could significantly increase the DR and expedite the bone healing procedure while increasing the osteogenesis quality [22, 26–29].

However, ACDO solution is a novel method and is yet to be used in human MRA due to limitations still present in the method. Different factors influence the DO process and limit the application of existing ACDO devices. There are still complications for using an ACDO device; more research and investigation need to be undertaken towards design and development of an ideal ACDO device for using in human MRA. In this review paper, developed ACDO devices for mandibular DO have been reviewed. In the following sections, designed and developed distractors, their working parameters, and technical specifications of developed systems have been introduced and discussed. Subsequently, current limitations in developed technologies have been addressed. At the end, for enabling an ideal ACDO treatment in human MRA while obtaining best possible results, existing gaps have been addressed and direction of future work have been suggested.

### Development of automatic continuous distraction osteogenesis devices

**Fig. 2:**
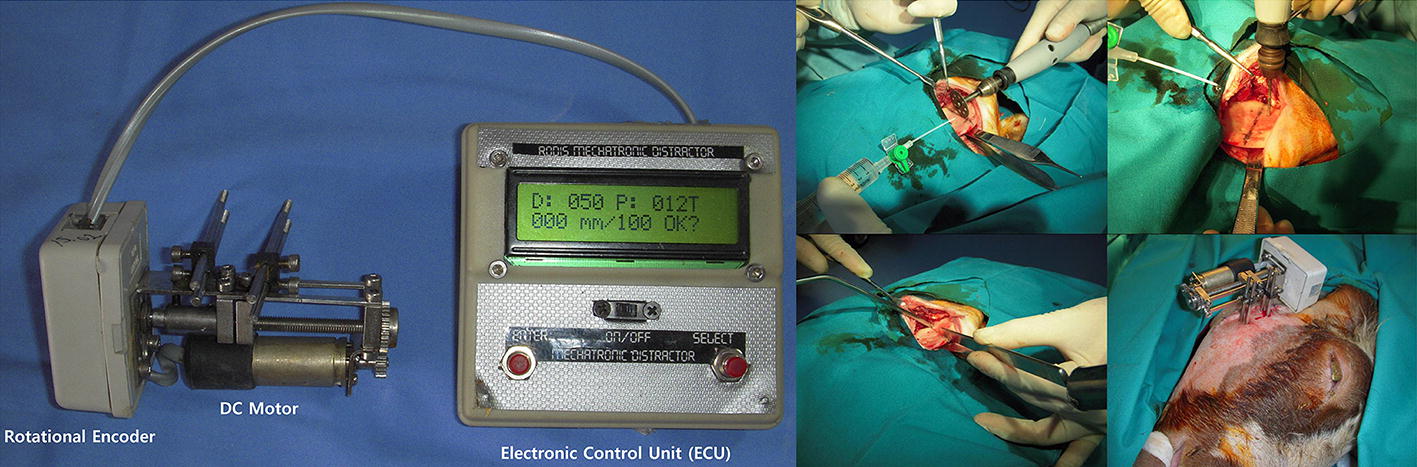
Extra-oral ACDO device with motor-driven system used in an animal study on sheep jaw bone (republished with permission of Wolters Kluwer Health Inc. Aykan et al. [1] https://journals.lww.com/jcraniofacialsurgery/Abstract/2014/07000/Mandibular_Distraction_Osteogenesis_With_Newly.95.aspx)

It is worth mentioning that ACDO is a novel solution in MRA; the application of ACDO devices for reconstruction applications has recently been emerging. Most performed studies have focused on developing various techniques, ex vivo models, and prototypes for achieving the best possible results and working parameters for automatically executing the DO protocol. During the last two decades, different ACDO devices based on various advanced manufacturing technologies for executing an automatic continuous movement on BS (based on standard characteristics of the DO protocol) have been designed and developed. Accordingly, experimental and animal studies have been performed to validate the functionality of the designed and developed techniques, as well as, evaluating the outcome of using an ACDO device in DO treatment. Similar to manual DO methods, there are two types of ACDO devices: extra- and intra-oral. In an extra-oral device, the distractor is placed outside the body on the defected zone and its mechanical connections are fixed to the bone while directly moving the BS from outside the body. Figure 2 illustrates an extra-oral ACDO device used in an experimental study on sheep.

**Fig. 3:**
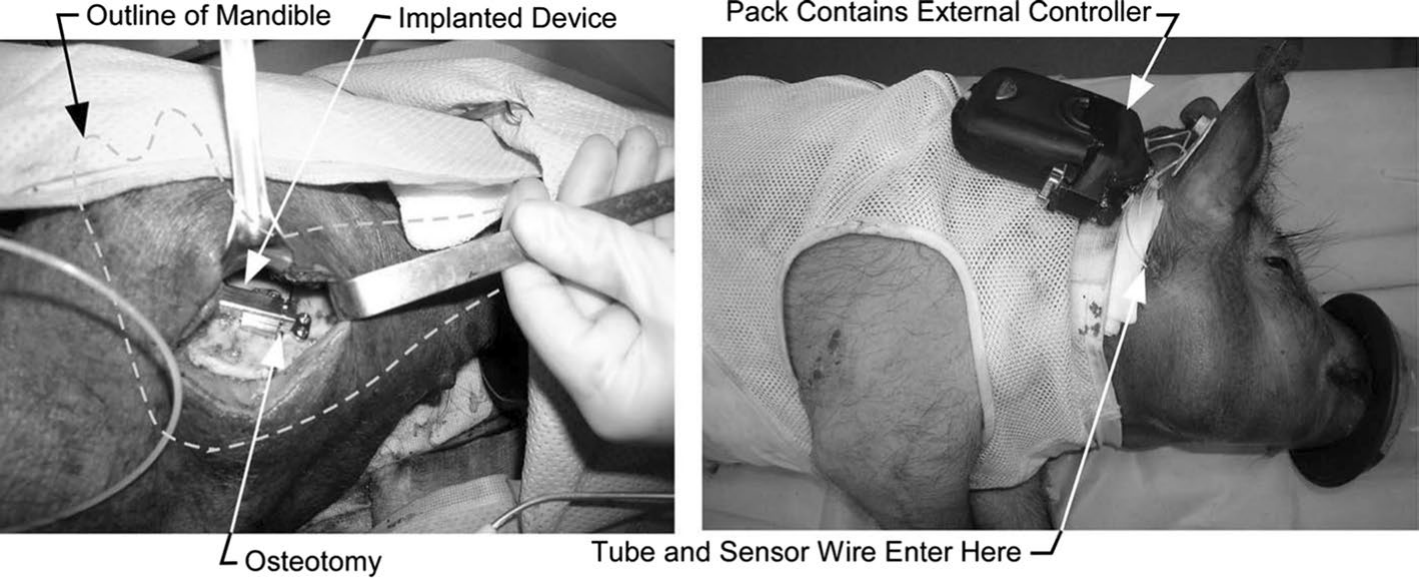
Implantable intra-oral ACDO device with hydraulic system used in an animal study on minipig (republished with permission of American Society of Mechanical Engineers ASME, from [30]; permission conveyed through Copyright Clearance Center, Inc.)

Similar to manual methods, in an extra-oral solution, major complications including scar formation, infections, and patient discomfort are involved during and after the DO procedure. Therefore, in recent research and developments, the tendency has moved towards developing intra-oral distractors. In an intra-oral solution, continuous DF is generated externally via an extracorporeal automatic system. By implementing a miniature transition system generated force is transferred to an implantable part of the device which is placed on the defected area, for moving the BS. Figure 3 illustrates an implantable intra-oral ACDO device with an extracorporeal controller used in an experimental study on minipig.

In both extra- and intra-oral ACDO devices, an automatic system is implemented for generating the desired continuous DF. In general, developed distractors could be categorized into two main groups according to their working principles. The first category of ACDO devices is motor-driven distractors, where an electrical motor combined with a precise mechanical structure is implemented to generate the desired DF. The other group is hydraulic distractors, whereby the device works based on hydraulic principles and the DF is generated through a hydraulic system.

### Motor-driven distractors

The first automatic motor-driven distractor was developed in 1999 by Ploder et al. [31] for mandibular bone tissue lengthening. In this preliminary study, an implantable intra-oral distractor for generating a continuous DF was designed and developed. In the experimental study on the sheep jaw bone model, the control unit and the power unit were inserted subcutaneously in the neck region of the sheep. The results of the performed study have shown the viability of such a novel ACDO solution. In addition, during experiments the device was well tolerated without difficulty. This distractor has the capability to move the BS with the accuracy of 0.04 mm/h with a DR of 1 mm/day. It is mentioned in the published work that there is the potential of decreasing the size of this mechanical system, while allowing a maximum stroke of 30 mm. In 2004, a feasibility study on a motor-driven mechanism for ACDO procedure was performed [32]. In this research, a prototype ACDO device was designed to validate that the requirements could be met. The main components in this mechanism include motor, lead screw, and transmission system. In this system a high-torque motor (Maxton RE10 brushed DC motor) was implemented to generate a continuous DF. The generated DF then goes through a translation system to translate rotary motion to linear movement. The motor’s shaft is connected to a lead screw via an impact coupling combination. Subsequently, the rotation of the motor and lead screw is translated to linear motion via a nut. This mechanism causes the BS moves in a linear path towards the destination, with a total size of 57 cm and maximum stroke of 15 mm. Furthermore, a set of bench test and analyses were performed to study the feasibility of the concept and prototyped mechanism. The device was able to generate a 70-N continuous DF. In 2008, a motor-driven high-rhythm automatic driver was developed to be attached to a custom-made or commercially available extra-oral distractor [21]. In the design of this system, a mini stepper motor is used for activating the system with a high rhythm, 8 distraction steps per second. The output of the motor is then transferred to the BS via a mechanical structure, which moves the BS towards the distention point while generating a DF of 19 N. In addition, a 1.5-V lithium button battery is implemented as the power source. This device was used in an animal study on rabbits, and the feasibility of the design and performance of the device were evaluated. The experiments on this device validated the superior outcome of the high-rhythm distraction compared to conventional DO treatment.

In 2010, a new motor-driven ACDO device was developed for mandibular reconstruction [33]. In this implantable battery system, a miniature DC motor-gearhead (series 0615, Faulhaber) is used and connected to a rotary-to-linear mechanism with average DF of 57 N and a maximum stroke of 15 mm. A PCB control circuit is also implemented for controlling the process. The device is able to generate a continuous DF and move forward the lead screw-head towards the destination. A digital electronics control system based on logic gates and digital ICs have been used in this ACDO device for controlling the positioning of the moving lead-screw head and transferring the BS to the desired location. In addition, by implementing an onboard 3.7-V lithium-ion battery, the necessary power for running the system and generating DF is provided. In 2011, a new extra-oral ACDO device by using a piezoelectric motor-based system was developed [20], to be used in jaw bone reconstruction applications by using DO treatment. In this device, a squiggle-type (SQL 3.4) piezoelectric motor is used for generating continuous DF. An AAT4900 chip is used in DC–DC step-up converter for driving the piezoelectric motor. In addition, an ATmega128 microcontroller is implemented for controlling the procedure, serial communication via RS-232 interface, and generating Pulse Width Modulation (PWM) signals. The designed software (based on Microsoft windows) processed the input data and sends the movement commands to the driver unit for driving the piezoelectric motor in intermitted and continuous modes. The developed device has a total size of 35 mm and a stroke distance of 7 mm. Performed experiments have shown that this distractor could generate a DF of 3 N. In another study in 2011, an auto-driven system with maximum DR of 3 mm/day for generating quasi-continuous DF was developed [34]. The device consists of a modified Seiko movement system with high output torque, a stepper motor, and a mechanical gearing system. An in vitro experiment was performed with using a customized system. During the experiment, the functionality of the system was evaluated, additionally, the output torque of the system with the amount of 4.268 × 10^−3^ kg m was measured and monitored.

In 2014, a new electromechanical extra-oral ACDO device for enabling continuous DO of mandible was developed [1], and the efficacy of the system was investigated on a sheep model. In this system a DC motor is used for generating the continuous DF. A rotational encoder is connected to the end of the DC motor’s shaft for measuring the rotational angle of the motor’s shaft subsequently, the position of the moving BS during the distraction procedure. An electronic control unit based on a microprocessor is designed and implemented within the system for controlling and driving the DC motor, as well as, measuring the position of the moving BS. Furthermore, investigations were performed to evaluate the performance of the device. In this animal study, five sheep underwent ACDO treatment and results have shown that with using an ACDO solution, the bone formation in new callus has enough quality and density while using an ACDO solution. In 2016, Kumar et al. [35] designed a motorized ACDO device and developed an automatic controller for using in the designed device. The developed controller is capable of controling an ACDO procedure with standard working parameters. In the control unit one microcontroller is implemented for controlling the process, two motor drivers are implemented for enabling driving 4 motors in dedicated axes (with an output power of 0.5 W), a Bluetooth module is used for enabling communication with the related application. In addition, by using four encoders, the position of each motor, and subsequently, the position of BS, could be determined. However, this research was limited to the development of the controller and more information on further developments of the design has not been reported.

In 2019, a high-precision intra-oral motor-driven ACDO device was developed [36, 37]. In this device, a linear control method, called Multi-Axis Automatic Controller [38, 39], is employed in the control unit to secure an accurate and stable positioning of the moving BS. In the control unit, an ATmega32 AVR microcontroller is implemented to control the ACDO procedure, communicate with the human machine interface (HMI) unit of the device, and driving the stepper motor. The HMI constitutes a liquid crystal display to visualize the process parameters during the treatment period. In addition, a keypad is connected to the control unit for setting and updating the process parameters. By implementing a serial eeprom, this device is capable of saving and load distraction data on an external flash memory, which enables recovering and continuing the distraction process in case a technical complication occurs. In this system, a miniature stepper motor and gear-box is implemented in the device which can provide an accurate shaft positioning, consequently, an accurate distraction. Miniature precision lead screws are used in the linear axis to enable a linear movement. A flexible miniature shielded transition system is connected to the extra-oral controller to transfer the generated DF to the installed implant on the jaw bone. Driving the system in micro-stepping drive mode provides a smooth distraction while moving the BS in a linear axis towards the destination position. The device is able to provide a distraction accuracy of 7.6 nm while producing a DF of about 35 N. Evaluations and experimental studies on a Ship mandible model validated the performance of this device. This high-precision technique for generating continuous DF is used in design of a laser-assisted ACDO device with the capability of distracting the BS in linear and non-linear distraction vectors. This device is capable of applying low-level laser therapy on distraction zone during the ACDO procedure via an implantable part [40]. In further developments on this system, le Roux et al. [41] designed a specific rechargeable battery system to be implemented with the device to make it portable; this battery system would enable using this device as a mobile distractor. Results of the experimental studies on the developed battery system have shown that this system could provide necessary power for running the ACDO device for 30 h before the need of being charged.

### Hydraulic distractors

In MRA hydraulic distractors are the second category of ACDO devices. In 2000, Keßler et al. [27] developed a microhydraulic ACDO device. In this system, an implantable intra-oral distractor is fixed subcutaneously as the intra-oral part of the device. An extracorporeal steering unit is used for controlling the thrust of the implanted distractor and distraction procedure. There are two major parts in the implantable intra-oral part: a cylinder and a piston, with a maximum stroke of 25 mm. In the extra-oral control unit, a hydro-pneumatic reservoir is connected to the thrust-control mechanism for applying a constant pressure on the hydraulic fluid and executing a mean pressure of 12 to 15 × 10^5^ (with a maximum pressure peak of 45 × 10^5^ Pa), while generating a perpendicular DF of 50 N for moving the BS. Subsequently, an animal study was performed on a minipig model for evaluating the outcome of continuous DO procedure compared to discontinuous solution. The results of this study have proved that using a DF for moving the BS could significantly improve the quality of the generated bone, as well as, the treatment time period. The first application in a human by using an ACDO device was performed in 2005 [42]; in this clinical study, Ayoub et al. reported the successful use of an implantable hydraulic ACDO device for lengthening the right ramus of a 65-year-old man in a DO treatment. The developed device consists of two main units: a mechanical intraoral implant placed on the distraction zone for moving the BS, and a portable external automatic hydraulic system with a battery-driven infusion pump for generating continuous DF of 20 N. A non-compressible drive cable is used in the mechanism to connect the mentioned units and transfer and pump the liquid to the implanted intra-oral distractor. Radiographical results have proved that using ACDO reconstruction technique in humans is viable, while providing promising outcome compared to conventional discontinuous DO techniques.

In 2009, a hydraulic ACDO device towards moving the BS in a curve-linear path via an implantable intra-oral actuator was developed [30]. This device has the capability to be controlled and set by user through a serial HMI. A spring-loaded supply reservoir and miniature valves are used in the design of this device. This system pressurizes the water and forces it into the hydraulic cylinder with a controllable flow. By using a high-pressure water tube, the generated DF is transferred to the implanted distractor placed on the bone in the distraction zone. In this device, a 3.3-V coin battery is used for running the control unit. In the control unit a PIC microcontroller is implemented for enabling communicate with the user HMI, controlling the solenoid valves, generating a real-time clock, and measuring the position sensor value by using analog to digital conversion feature of the microcontroller. In addition, a 12-V power supply is used in the design of the device to provide the necessary power for running the system. Two solenoid valves are implemented and under control of control unit. This combination could successfully enable ACDO procedure with a DR of 1 mm/day while generating an average DF of 25 N and a peak load of 40 N, in a maximum stroke of 25 mm. Subsequently, the device was used in an animal study on 2 groups of several minipigs, for reconstructing a bone defect in the maxillofacial area, and lengthening the skeletal bone tissue by using ACDO solution. In this experiment the influencing factors on ACDO treatment were evaluated. Results of this study have shown the effectiveness of ACDO method compared to conventional discontinuous DO methods. In another study in 2009, Djasim et al. [22] performed an investigation on continuous DO of the nasal bones in a rabbit model by using a custom-made hydraulic continuous distractor. The purpose of this study was to evaluate the performance and outcome of the ACDO solution compared to conventional discontinuous DO solutions with regard to the bone regeneration. In this study an extra-oral hydraulic distractor was custom-made to fit the nasal bone of the animal model. The system consists of two cylinders, a connection for a catheter to transmit the fluid, and an external control unit. In the control unit a microprocessor is implemented to a syringe pump while generating a DR of 0.9 mm/day with a total stroke of 15 mm.

In 2013, through further developments on hydraulic distractors, Peacock et al. [13] designed and developed a new intra-oral hydraulic ACDO device with the capability of generating DR higher than 1 mm/day. The goal was to determine if ACDO with DRs greater than 1 mm/day results in bone formation with sufficient osteogenesis quality. This system consists of an extracorporeal control unit which is placed outside of the body, and an implantable distractor which is installed on the distraction zone. In the control unit a microcontroller-based circuit with position-feedback is used for controlling the implemented hydraulic distractor and executing DF for moving the BS. A spring-powered hydraulic reservoir is implemented and supplies 2 × 10^6^ Pa at full spring extension and 3.4 × 10^6^ Pa at full spring compression for pressurizing the water to the implanted distractor through a micro-dispensing solenoid valve. Therefore, this system could provide 25 to 40 N. Furthermore, results of experimental and animal studies on minipigs with using various DRs up to 4.5 mm/day have revealed that by using a continuous DF in an ACDO solution, higher rates of distraction, as well as, better bone formation could be successfully achieved. In 2015, this research group published the results of using this ACDO device in an animal study on minipig model [28] for demonstrating that ACDO is an effective solution to achieve clinically relevant lengthening. In this study, a modified version of this device with a DR of 3 mm/day and a total stroke of 30 mm was used.

### Evaluation of designed and developed automatic continuous distractors

DO is a novel bone lengthening technique in MRA. In further developments on this method, recently ACDO devices have been introduced to be used in mandibular DO treatment, as an automatic solution with superior outcome compared to conventional manual DO methods. Different ACDO devices, prototypes, models, and simulations have been designed, developed, and used for evaluating and validating this novel technique, as well as, enabling using the ACDO solution in human MRA. Different efforts have been undertaken for improving the accuracy of such automatic systems, safety of distractor, size, and treatment period; acceptable outcomes have justified the developed techniques from different technical and physiological aspects. However, the application of produced ACDO devices has been limited to experimental and animal studies. More research and investigation need to be conducted towards an ideal design of an automatic continuous distractor for using in human MRA. In the following subsections biological and physiological aspects of using ACDO devices, characteristics of developed ACDO devices and required standards, the aspect of power consumption, and current limitations have been discussed.

### Continuous distraction of the bone: proof of principle

DO as an endogenous tissue engineering technique that has been widely proved successful in animal and clinical studies on human MRA. The DO technique enables the new bone formation by applying callus healing mechanism [43]. Results of experimental and animal studies have validated that by using an ACDO treatment, the regenerated bone tissue is well-formed and the formed tissue has enough quality [13, 28, 44]. In one of the first studies to use DO on a sheep model [31], it was shown that both cartilaginous and/or membranous bone formation could be observed in the tested animals by using an implanted motor-driven device. In this study, the bone formation was confirmed in the tested animals by radiological and histological evaluation. Later in 2001, the application of a continuous lengthening device, during a DO procedure on sheep’s mandible, was proved to be satisfactory [45]. In addition, in another study in 2000, Keßler et al. [27] by an animal study on minipigs showed that the hydraulic bone distractor can be used without any problems and complications in a successful DO treatment. The first case of testing the automatic continuous distraction osteogenesis on humans was reported successful in 2005 [42]. The patient had refused to use the grafting which is the standard procedure in this case. Therefore, the surgeons decided to lengthen the vertical height of the patient’s mandible by using a hydraulic ACDO device. The results of this clinical study have shown the successful outcome of applying ACDO on a human mandible. In 2008, a motor-driven device was used for automatically lengthening the BS for filling the mandibular defect in rabbit model [21]. It has been revealed via histology, radiography and micro-computerized tomography that the process has been successful. In 2009, a miniature high-pressure hydraulic DO device was used in a study on porcine cadaver head and in live pigs [30]. The test sequence of this study showed successful results. The efficacy of continuous DO compared to a discontinuous DO was tested by Djasim et al. [8]. In a study on rabbit nasal bones, they found that in an ultrasonographic radiographic and microcomputed tomographic evaluations, continuous DO showed better results compared to discontinuous DO. The next study evaluated the results of continuous and discontinuous DO was performed on minipigs by Peacock et al. [13]. They evaluated the speed of DO and found that continuous DO at 1.5 and 3.0 mm/day rates had shown better bone formation compared to discontinuous DO at rates faster than 1 mm/day. In a later study, the skeletal and soft tissue response to ACDO solution was evaluated in a minipig model [44]. It was found that a faster rate (1.5 and 3 mm/day) with automated DO is comparable to slower rates by discontinuous DO. In 2015, the same research group [28], showed that bilateral application of a curvilinear, continuous and automated DO is effective at rate of up to 3 mm/day using a minipig model. In 2014, a continuous DO device was developed and tested on a sheep mandible bone [1]. Macroscopic and radiologic evaluations have shown that the callus was well formed. Therefore, from obtained results it could be deduced that continuous distraction of the bone could provide better results with regard to bone tissue regeneration compared to discontinuous manual methods.

### Automatic continuous distractors and required characteristics

There are key elements significantly influencing a continuous DO procedure: the rate and the rhythm of distraction, the generated DF, and the distraction vector are from those major factors influence the outcome of the treatment [20, 22, 46]. To achieve a successful treatment and high-quality formed bone tissue by using an ACDO solution, the device should cover all required parameters of a standard DO protocol. In MRA the DO technique is usually used for reconstruction of the mandible, alveolar, mid-face, and cranio-orbit bones. Studies have shown that a standard DO protocol for reconstructing different cranio-maxillofacial areas require the following working parameters: Distraction length: 10–20 mm. DR: 1–3 mm/day. Minimum continuous force: 35 N. Execution time: 7–10 days [16, 21, 27, 29].

The general characteristics of developed ACDO devices are summarized in Table 1. The most accurate hydraulic device can generate a maximum distraction accuracy of 10 μm/step with a mean step error of 86 μm per step [30], where motor-driven devices could generate a maximum distraction accuracy of 7.6 nm per step with a mean step error of 0.06 nm per step [36]. Using high-precision motor and gear-box combination in motor-driven systems has a much better performance in generating movement with a high-precision accuracy with very low movement error. Therefore, it can be deduced from Table 1 that motor-driven devices have a much more accurate distraction accuracy for moving the BS. In addition, the ACDO device should be able to generate a minimum DF of 35 N for moving the BS [25, 44, 47–49]; only a few ACDO devices could successfully generate a smooth and sufficient amount of continuous force for moving the BS. It has shown in Table 1 that existing distractors could successfully distract the BS with a DR up to 5 mm/day. Therefore, developed devices have enough DR, DF, and distraction accuracy to be used in a standard ACDO treatment. In addition, the size of ACDO device, including the external unit and the internal implantable distractor, is another important aspect in development of ACDO devices and significantly affect the outcome of DO procedure. Reducing the total size of the implantable distractor could reduce tissue damage, infections, and bone fracture, as well as, minimizing the side effects of such a treatment on human body [20, 23, 30]. In novel designs of ACDO devices, the tendency is to develop a miniaturized implantable distractor for installing on the defected zone for subcutaneous and submucosal applications especially in unfavorable anatomical zones, i.e., in the midface [20, 27].

**Table 1:** The general characteristics of developed ACDO devices

Year	Refs.	Mechanism	Total size (mm)	Maximum travel (mm)	Generated force (N)	Operated DR (mm/day)	Distraction accuracy (μm)	Distraction step error (μm)
1999	[31]	Motor-driven	–	13.6	–	1	40	0.5
2000	[27]	Hydraulic	–	–	30 to 50	1.5	–	–
2004	[32]	Motor-driven	57	15	70	1	1000	20
2005	[42]	Hydraulic	–	16	20	1	–	–
2008	[21]	Motor-driven	55	10	19	0.9	–	80
2009	[30]	Hydraulic	30 to 100	25	25 to 40	1	10	86
2009	[22]	Hydraulic	–	15	–	0.9	–	21,000
2010	[33]	Motor-driven	60	15	57	1	600	–
2011	[20]	Motor-driven	35	7	2.84	1.4	200	2000
2011	[34]	Motor-driven	–	3	–	3	0.75	30
2013	[13]	Hydraulic	18	12	25 to 40	1.5	–	Average < 500
						3		Average < 1000
						4.5		–
2014	[1]	Motor-driven	–	18	–	2.4	300	4
2015	[28]	Hydraulic	–	30	25 to 40	3	–	–
2019	[36]	Motor-driven	25	22	38	1	0.0076	0.00006
						3		0.00006
						5		0.00002

**Fig. 4:**
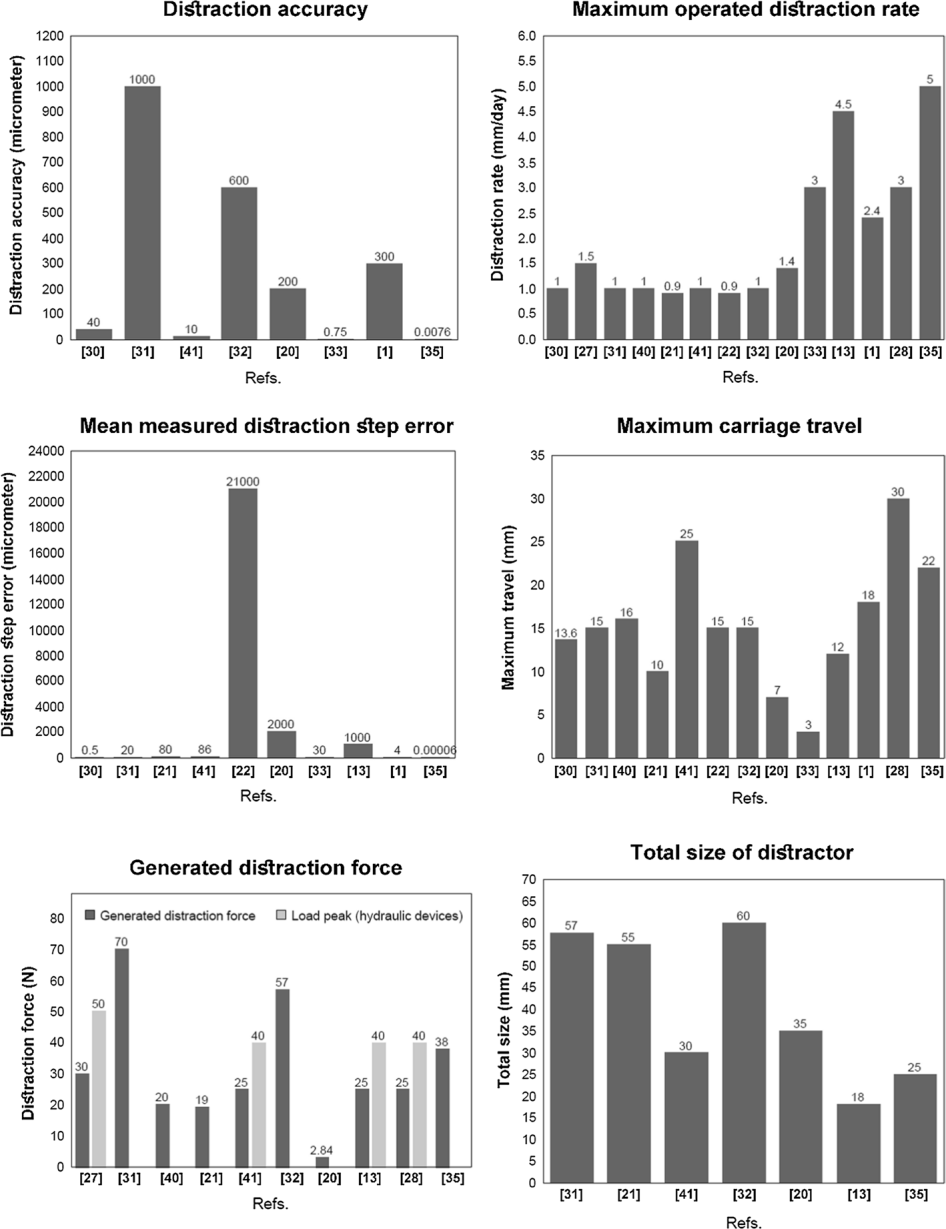
Comparing major specifications of developed ACDO devices

According to the specifications and working parameters of developed ACDO devices, by using data mentioned in Table 1, graphs have been generated for comparing characteristics of ACDO devices, as illustrated in Fig. 4. It could be deduced from the graphs that [36] has the maximum distraction accuracy of 0.0076 Âµm, maximum operated distraction rate of 5 mm/day, and minimum distraction step error of 0.00006 Âµm. [28] has a maximum carriage travel of 30 mm. According to the performed studies, the minimum DF is about 35 N [1, 13, 27, 30, 32, 33, 36] and could generate a sufficient DF, where [36] with generating a DF of 38 N has the minimum difference with the ideal amount of DF (35 N). In addition, [13] has the minimum size (18 mm) among all developed devices.

### Continuous force generation

Continuous force generation is the most important aspect in the ACDO procedure. DF is the most important factor influencing the quality of outcome in a DO treatment and affect the bone regeneration and consolidation results. Beside the fact that the generated DF should have a minimum amount of 35.6 N (4.2 N.cm) for moving the BS [33], the force generation significantly affect the distraction process in different capacities. Different systems have been developed and used in ACDO devices for generating the desired DF. The developed distractor based on piezoelectric motor [20] could generate a DF of 3 N, which is suitable for animal studies on small-size animal models such as a rat and mouse. Thus, for providing the required DF for human MRA, this solution is limited in sufficient DF generation.

**Fig. 5:**
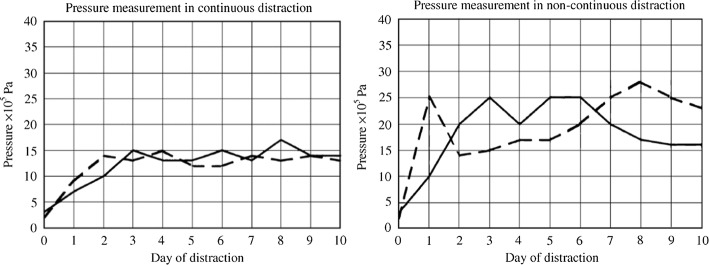
Pressure curve in ACDO procedure (left) and non-continuous DO procedure (right) (reprinted from Keßler et al. [27] copyright (2000), with permission from Elsevier)

In hydraulic ACDO devices, although the generated pressure is enough to execute the BS movement with required DF, due to masticatory action, considerable variations in generated DF are monitored [13, 27, 30]. This effect is illustrated in Fig. 5; it has been shown that the generated pressure for executing the DF for moving the BS, in both continuous and discontinuous methods of distraction have an initial peak value while executing the DF. In the performed studies and analyses on hydraulic ACDO devices although in a hydraulic distractor a mean value of 20 × 10^5^ Pa is required for generating the necessary DF, pressure peaks up to 50 × 10^5^ Pa have been reported [27].

**Fig. 6:**
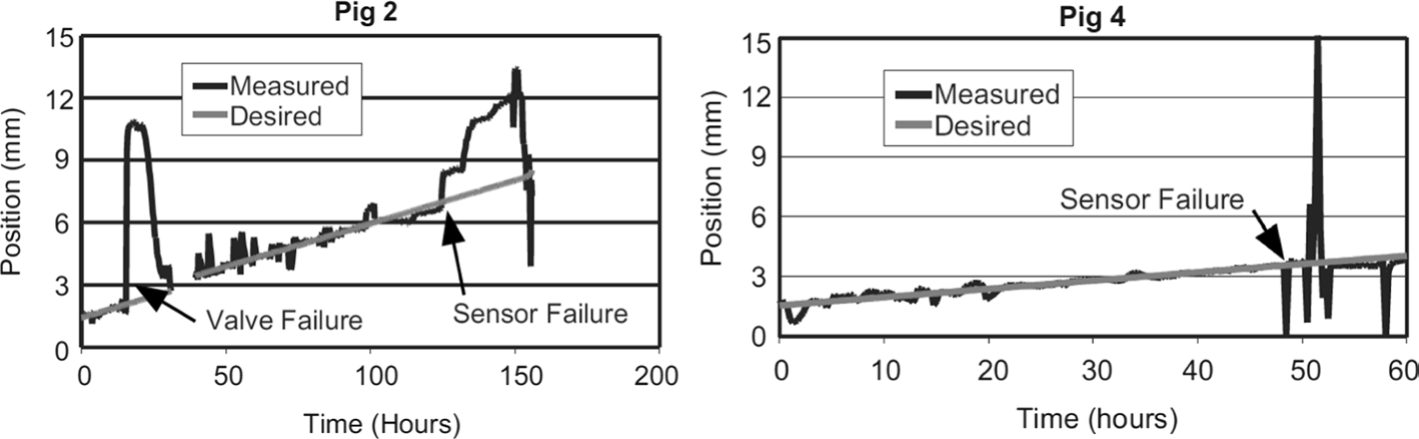
Pressure measurement during ACDO procedure by using a hydraulic distractor (republished with permission of American Society of Mechanical Engineers ASME, from [30], Copyright Clearance Center, Inc.)

In addition, Fig. 6 illustrates the results obtained in the performed animal study by using a hydraulic ACDO device [30], besides the sensor errors and value errors of the developed system during the performed animal study, the effect of load peaks in each activation sequence of the hydraulic system can be seen in the measured data during the process.

**Fig. 7:**
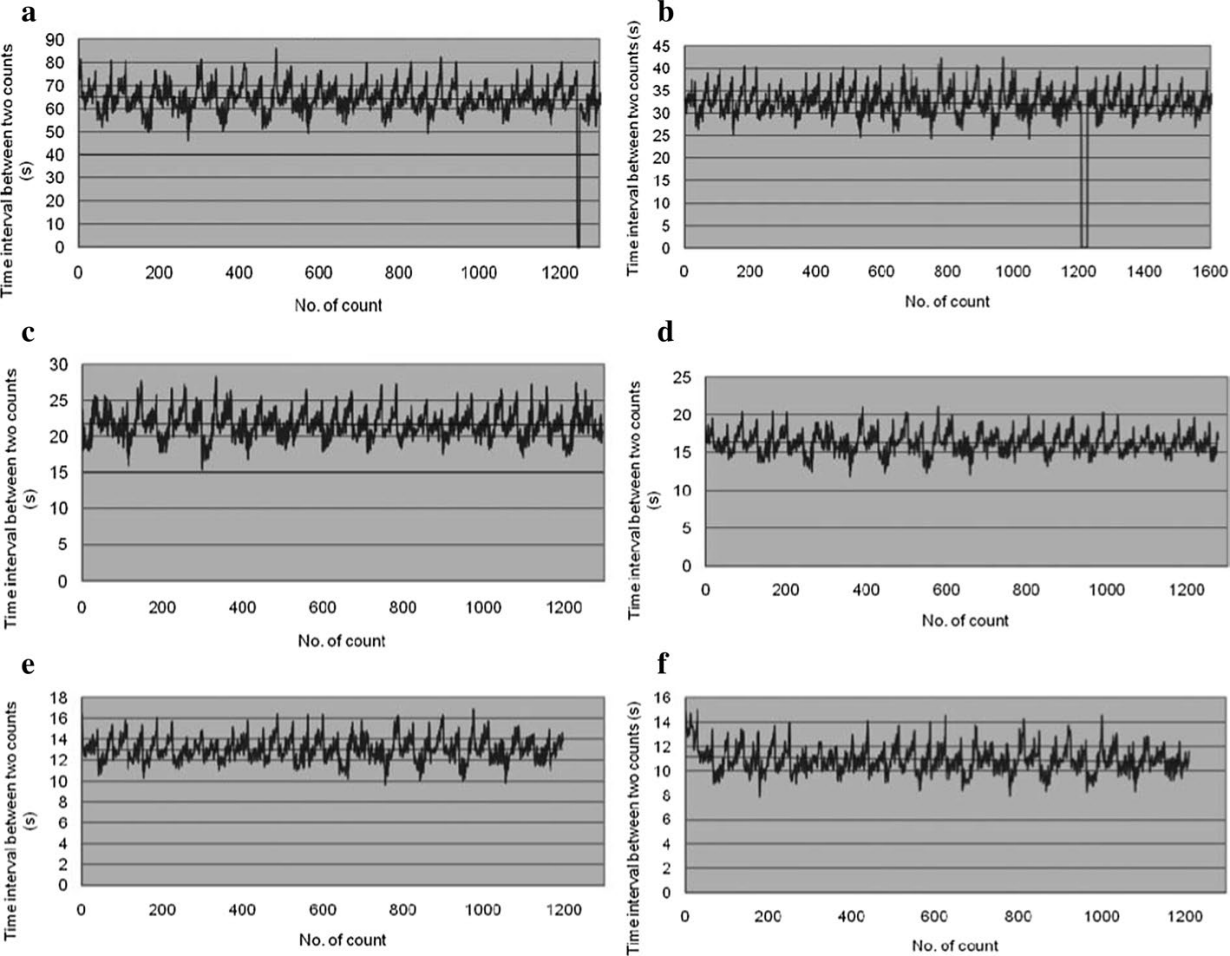
Time interval between each pulse versus angular displacement of the distractor with designed speed at 3.0 mm/day (**a**), 2.5 mm/day (**b**), 2.0 mm/day (**c**), 1.5 mm/day (**d**), 1.0 mm/day (**e**), and 0.5 mm/day (**f**) (Zheng et al. [34], copyright © 2011 by (SAGE publications). Reprinted by permission of SAGE publications, Inc.)

In an experimental study, by using a quasi-continuous motor-driven ACDO device in different test procedures, the time interval between each distraction step versus angular displacement of the distractor was measured while applying various DRs by varying the distraction rhythm [34]. In this study, six different DRs from 0.5 to 3 mm per day were executed in the performed experiments. The measured data during these experiments are illustrated in Fig. 7. It has shown that increasing the DR would result in a smoother distraction. The waveform representing the angular displacement in graph A with DR of 3 mm/day compared to the waveform representing the angular displacement of distractor in graph F with DR of 0.5 mm/day shows the influence of increasing the DR on the generated ripples and noises while driving the stepper motor of the system and moving the BS. In addition, by increasing the step accuracy of the distractor, higher rates of distraction could be implemented in recent animal studies successful ACDO treatments by using DR up to 4.5 mm/day have been reported [13].

**Fig. 8:**
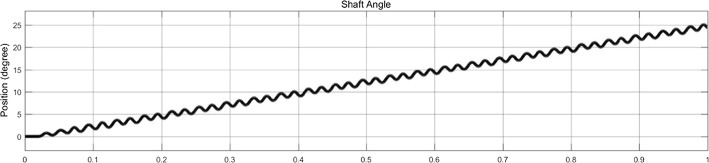
Shaft position during ACDO procedure with using a motor-driven device [36]

**Fig. 9:**
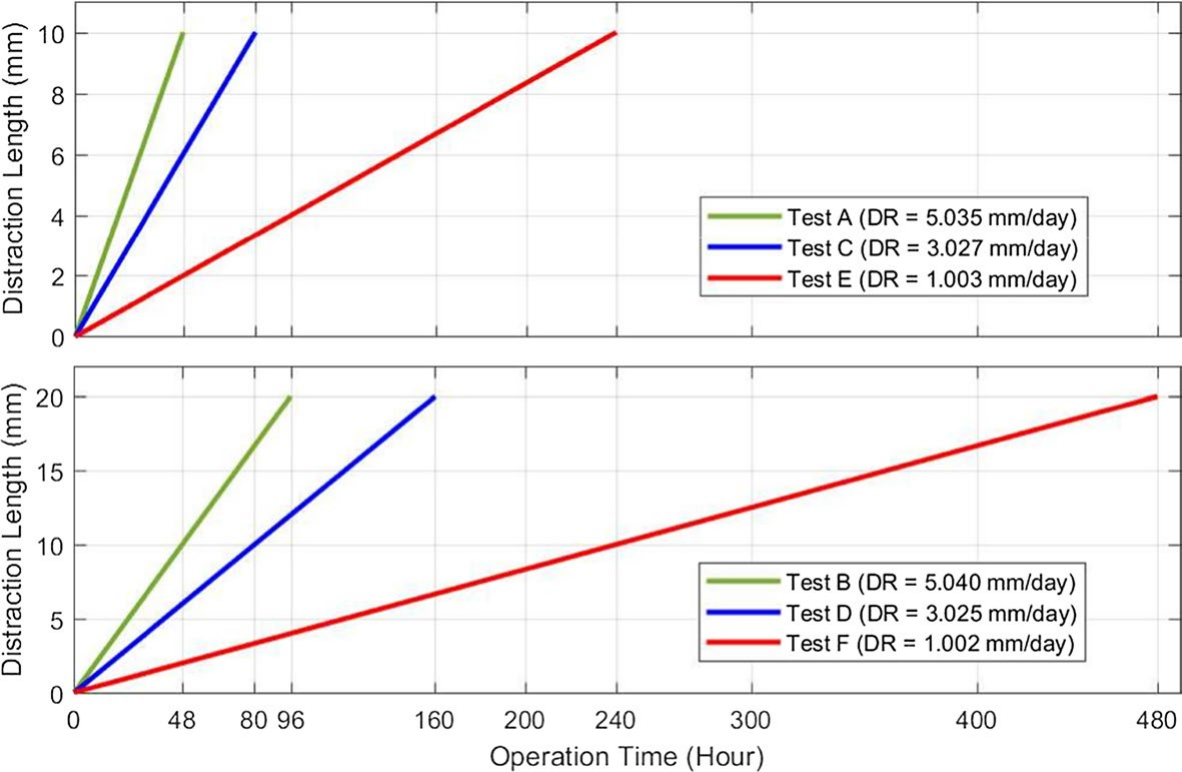
The mean measured distraction length and distraction rate in different conditions of distraction osteogenesis with using a motor-driven device [36]

Motor-driven ACDO devices have shown promising results in continuous force generation. Using electrical motor and mechanical gear combination in a precision mechatronic system could enable generating a smooth and stable force for moving the BS; in a motor-driven system there is no pressure peak while executing the continuous DF. In a recent study, by using a high-precision motor-driven linear system, a stable DF of 38 N for moving the BF was successfully generated [36]. Figure 8 illustrates the shaft position of the implemented mini stepper motor and gear-box in this ACDO device in a DO procedure with distraction accuracy of 7.6 nm/step and DR of 5 mm/day. It can be deduced from Fig. 8 that the device is capable of executing a high-precision movement of the BS while generating a very smooth and sufficient DF. Figure 9 illustrates the mean measured distraction length and DR in different conditions of distraction osteogenesis with this device. The results of tests have shown that this distractor could successfully complete DO procedures with different DRs, while the BS is moving in a linear manner with a smooth continuous force towards the desired position.

To be able to use an ACDO device in a successful DO treatment in human MRA, the distractor needs to generate a smooth and sufficient DF, not to damage the tension-sensitive structure of the distraction zone [27]. High peaks during continuous force generation are problematic and influence the osteogenesis quality and consolidating process. Mentioned disadvantage and limitations in continuous force generation could significantly affect the bone healing results and influence the outcome of the treatment. Therefore, from this point of view, using motor-driven ACDO devices in a DO procedure would result in generation of a high-quality, stable, and smooth continuous DF for achieving best possible results during the treatment.

### Aspect of power consumption

The goal is to design and develop an ideal ACDO device to be used in human MRA. One of important aspects is to make the device portable such that it could be used in clinical applications in humans. The aspect of power consumption is important and thus the required power for running the system should be calculated. According to the standard DO protocol, the device needs to work continuously for an activation phase of between 3 and 10 days [16, 21, 27, 29]. Therefore, suitable batteries and rechargeable battery systems need to be designed and implemented within the development of the device, and the working span before the need of a charging process is to be calculated. Subsequently, optimal charging conditions need to be determined and ensuring that the necessary power is provided for the ACDO device should be considered. The power consumption of the system is an important factor that influences and limits features of the device. For example, when the device uses a high amount of power, more powerful batteries with higher capacity need to be implemented in the device.

Among all previously developed ACDO systems, a few battery systems have been designed and developed. There are various significant factors which provide room for comparison in the design of the systems. Tables 2 and 3 represent the relevant information about the previously developed ACDO systems which include power supply design. Table 2 lists the power requirements of the developed ACDO systems. Table 3 indicates the characteristics of the developed power systems. The data presented in these tables are presented as per literature and it is thus important to note that in some published works not all the necessary information about the battery systems have been indicated. The dominant characteristics of the power systems presented in literature which will be compared include the power supply required by the designed system, the time for which the system is to operate, the size constraints of the system and whether it is desired for the entire system (including the power supply) to be implantable. These factors largely influence the battery cell technology which is chosen and the time for which the power system can operate before being depleted.

**Table 2:** System power requirements of previously developed devices presented in literature

Refs.	Year	Required voltage (V)	Current (A)	Energy (Wh)	Power (W)
[31]	1999	3.6	–	–	–
[32]	2004	6–12	29 × 10^−2^	0.223	1.75
[21]	2008	1.5	–	–	–
[33]	2010	3.7	63.858 × 10^−3^	0.266	0.24
[35]	2016	3–12	15 × 10^−3^	–	0.5
[41]	2019	5	16 × 10^−2^	192	0.8

**Table 3:** Characteristics of developed power supplies for mandibular distraction osteogenesis devices

Refs.	Year	Battery technology	Nominal voltage (V)	Voltage supply (V)	Capacity	Implantable	Rechargeable
[31]	1999	Lithium-ion	3.6	3.6	–	No	Yes
[32]	2004	Lithium–silver Vanadium oxide	6.4–13.6	6–12	–	No	–
[21]	2008	Lithium button	1.5	1.5	–	Yes	No
[33]	2010	Lithium-ion polymer	3.7	3.7	–	Yes	–
[35]	2016	Lithium-ion	7.2	5–7.8	–	No	Yes
[41]	2019	Lithium-ion cells	7.2	5	4.8	No	Yes

For the device designed by Crane et al. [32], the voltage requirements are suggested between 6 and 12 V, and the motor which is suggested to be implemented is a Maxon RE10 DC motor. It has been calculated that the maximum continuous current required by the motor will be 290 mA if a voltage of 6 V is supplied; the required power is thus calculated to be 1740 mW. Different operating times are provided according to different load and supply voltage options, the required energy is thus calculated according to the longest operating time when utilizing a 6-V power supply, resulting in a 0.223-Wh requirement. Chung et al. [33] developed a system which utilized a power system with a nominal voltage of 1.2–3.7 V. The system consisted of a commercial Sc motor with a planetary gearhead where the motor which was used was a series 0615, Faulhaber MicroMo motor. The system was thus calculated to have a maximum energy requirement of 0.266 Wh and power consumption of 0.24 W. The battery system which was designed in [41] was specifically designed for the specifications presented in [36]. By referring to Table 2, it could be seen that the design requirements of the required power system in this device are a supply voltage of 3–12 V with a power consumption of 0.5 W. As illustrated in Table 2, the power system developed in [41] met these design requirements by providing a nominal voltage of 5 V and 0.8 W. The rest of the literature did not provide sufficient information to calculate the energy requirements and thus these values are not indicated within Table 2. The rest of the literature did not provide sufficient information to calculate the energy requirements and thus these values are not indicated within Table 2.

The most common trend found within the developed power systems is the correlation between whether the system is implantable and rechargeable, also influencing the type of battery technology which is chosen. As illustrated in Table 3, all the systems were powered by Lithium-Ion battery technology. This is due to the high energy density of Lithium-Ion battery technology, making it suitable for the application. As illustrated within Table 2, the power systems were designed based on whether they were to be implanted. Lithium-Ion battery technology is predominantly a rechargeable battery technology, and thus upon designing the power systems, it was required that the designer consider the power capabilities of the batteries. If the entire system, including the power supply, was to be implantable, it would not be possible to recharge the power system during operation and the battery was thus required to provide energy for the entire distraction process. This is indicated by [21] and [33], whereby the system was implantable, and one was thus not able to recharge it during the power supply during the distraction process. In contrast, the system developed by le Roux et al. [41] did not require for the power supply to be implantable could thus be recharged during operation. In this system, the time for which the system could operate was 30 h. In the work presented by Crane et al. [32], the efficiency of the developed system was tested under various operating conditions, with the highest efficiency of the power supply system found to be when the system was operating at a supply voltage of 6 V and the supplied load for the DO procedure was 19.8 N. Experimental tests for the operating time and operation of the power supply was not indicated within any of the other literature. Experimental testing of a Lithium-Ion battery prior to implementation is vital to ensure that all the system requirements are met and that no safety issues are encountered.

Monitoring of the battery system, especially if it is a lithium-ion battery system is an important aspect of the operation of the lithium-ion batteries for distinguishing if the battery has reached its lower voltage limit upon discharge, ensuring that the system does not heat up excessively, and ensuring that there is not an imbalance in the cells if more than one cell is used for the power supply. In the work completed by Ploder et al. [31], it is indicated that a buzzer makes a sound when the lower voltage limit of the battery is reached, such that the battery will not operate below its threshold voltage. This is particularly important in implantable devices as it is vital to sure that no risks are posed which could harm the patient in any way. In [41], a battery management system is implemented which consistently monitors and controls the battery, shutting it off if any errors are encountered.

Development of ACDO devices involves many aspects and it is important that the power supply is considered carefully when developing a device. There are many factors which play an important role in the operation of the device, not only ensuring that the ACDO is provided with sufficient power during the DO procedure, but also ensuring that it is done in the safest and least invasive manner. It is thus suggested that developers consider all the necessary aspects when developing a power system for medical devices, ensuring that the necessary battery tests are conducted such that the developer is confident that the system not only meets the power requirements, but does not experience excessive heating, current or voltage spikes. Testing of the battery system prior to usage also ensures that the battery or cell is in working order.

## Current limitations

In last two decades ACDO devices have been designed and developed. Main influencing factors in such a novel limb lengthening technique have been studied and discovered. Different distractors have been designed and developed to validate the viability of this new reconstruction technique to be used in human MRA in the future. Studies and published works have proved that the developed techniques can successfully cover all requirements of a standard DO treatment protocol. In addition, various experiments and animal studies have proven that using a continuous force for moving the BS during a DO treatment can improve the outcome and improve the quality of generated bone tissue. However, developed ACDO devices have been limited to experimental and animal studies thus far. Further improvements need to be undertaken for developing an ultimately suitable ACDO device, to be used in human MRA.

Extra-oral ACDO devices have shown successful results in providing required working factors during treatment while achieving successful outcome after the procedure. However, there are serious complications and limitations that limit the application of extra-oral devices in human MRA. Scar formation, infections, and patient discomfort are among the challenges that still exist. Exposure of the extra-oral components in an extra-oral solution is problematic. Using an intra-oral solution would result in less soft-tissue injuries, scar formation, and complications during and after the treatment period [20]. Recent research has shown a larger focus on the development of intra-oral distractors. An intra-oral ACDO device including an implantable mechanical part inside the body would be a superior choice compared to extra-oral ACDO devices.

In the performed studies, motor-driven systems have shown less complications and errors during their performance. However, both categories of ACDO devices, hydraulic and motor-driven, have their own disadvantages and limitations due to the origin of the implemented techniques. Hydraulic systems generate a pick force at the beginning of the execution of DF, which could significantly damage the bone tissue during the soft and continuous distraction of the BS. Also, the size of the extra-oral unit and the implantable part of hydraulic devices could potentially cause complications in such an ACDO treatment. Power consumption of hydraulic devices is also another issue in developing portable devices with long-lasting battery life. Alternatively, motor-driven systems have problem in DF transition. Using a mechanical and miniaturized transition system for transferring the generated force to the implanted part of the device could be problematic during the DO treatment. Therefore, both categories of ACDO devices have limitations and are yet to be used in an ideal ACDO procedure for human MRA.

In addition, portability of the device, total size, power management systems, safety sensors, and rechargeable power supply are other important parameters that need to be considered in future developments of ACDO devices. Finally, more research and investigation need to be conducted for developing an ultimate ACDO device which enables this novel treatment method to be used in a successful and safe human MRA.

## Conclusions

DO is a method of regenerating new bone tissue with using gradual supply of tensile stress across the osteotomized site. Using a continuous solution in DO treatment would result in accelerated osteogenesis and superior bone-fill score compared to discontinuous DO solutions. In addition, automatic continuous distraction offers more advantages, including less pain and enhancement in the quality of generated bone tissue during the treatment. Despite the fact that the ACDO method has shown superior results and is considered as a reconstruction solution with best achievable results thus far, this solution still presents limitations to the application in human MRA. Recently developed devices have shown promising results and fulfill all technical aspects of a standard DO procedure for a successful treatment. However, there are different complications which limit the application of the developed ACDO devices to animal models and experimental tests. More research and investigation need to be undertaken towards the design and development of an ideal ACDO device to be used in human MRA, especially to maximize the reliability of the device, safety, portability, and size.

With respect to recent contributions, technical and required standard working factors are fully covered via developed technologies. Therefore, future research and developments should be concentrated on decreasing the size of the intra-oral part of the device, the material, safety, and portability of the device. The ultimate outcome would be designing and developing an implantable intra-oral distractor connected to a mobile extracorporeal battery-based control unit. In addition, providing a wireless communication between the HMI unit and the control unit would reduce the size of controller and improve the functionality of the device.

## Data Availability

The research data related to this review paper are included within the article. For more information on the data, contact the corresponding author.

## Abbreviations

DO: Distraction osteogenesis
MRA: Maxillofacial reconstruction applications
BS: Bone segment
DF: Distraction force
DR: Distraction rate
ACDO: Automatic continuous distraction osteogenesis
PWM: Pulse width modulation
HMI: Human machine interface
